# Causes of Emergency Department Admission and Outcomes Among Hematologic Versus Solid Tumor Patients Admitted to a Palliative Care Unit

**DOI:** 10.7759/cureus.117515

**Published:** 2026-10-05

**Authors:** Waleed A Bokhari, Sumaia A Felimban, Sara M Aljunaid

**Affiliations:** 1 Department of Palliative Care, King Abdulaziz Medical City, Jeddah, SAU

**Keywords:** emergency department, hematologic malignancies, inpatient palliative care, palliative care, short-term outcomes, solid tumors

## Abstract

Background: Patients with hematologic malignancies frequently receive more aggressive end-of-life care and are less likely to receive timely palliative care than those with solid tumors. There is still little data, though, comparing the short-term outcomes of patients later admitted to inpatient palliative care units with their emergency department (ED) presentations. In this study, patients with solid tumors and those with hematologic malignancies were compared in terms of short-term clinical outcomes and the reasons for ED admission.

Methods: A retrospective observational study was carried out on adult patients who were admitted straight from the ED to an inpatient palliative care unit. Patients were divided into groups for solid tumors and hematologic malignancies. Clinical, disease-related, and demographic information was taken from electronic health records. We compared the two groups' reasons for ED admission, hospital diagnoses, palliative care interventions, 30-day mortality, and 30-day readmission. To find independent predictors of 30-day mortality, multivariable logistic regression analysis was carried out.

Results: Of the 256 patients, 233 (91. 0%) had solid tumors, and 23 (9. 0%) had hematologic malignancies. While patients with solid tumors were more likely to have metastatic disease (all p < 0.05), patients with hematologic malignancies were significantly older and more likely to be diagnosed with recurrent or relapsed disease. Regarding the reasons for ED admission, ED disposition, final hospital diagnoses, palliative procedures, 30-day mortality, and 30-day readmission, there were no discernible differences between the two groups. Malignancy type was not independently linked to a higher 30-day mortality in multivariable analysis, but increasing age and severe symptom burden at ED presentation were.

Conclusions: Patients with solid tumors and hematologic malignancies had similar reasons for ED admission and similar short-term outcomes following admission to an inpatient palliative care unit, despite differences in baseline disease characteristics. Age and symptom severity were independent predictors of 30-day mortality, suggesting that clinical status at presentation may be more relevant than the type of malignancy in predicting short-term outcomes.

## Introduction

Palliative care, which aims to improve quality of life by preventing and relieving patients' and their caregivers' physical, psychological, social, and spiritual suffering, has become a crucial part of comprehensive cancer care. There is mounting evidence that early palliative care intSkipegration with disease-directed treatment improves communication, symptom management, and end-of-life care planning [1, 2]. Palliative care utilization varies among cancer populations despite these advancements. Compared to patients with solid tumors, patients with hematologic malignancies consistently have lower rates of palliative care referrals and are more likely to receive aggressive treatments close to the end of their lives [3-5]. Unpredictable disease trajectories, continued reliance on blood product support, the lack of obvious signs indicating the shift to end-of-life care, and hematologists' reluctance to start conversations about prognosis, hospice referral, and care objectives are some of the factors that contribute to these disparities. Evidence supporting the integration of early palliative care in hematologic malignancies has only recently started to surface, despite the long-established benefits for patients with solid tumors. Prior research has demonstrated that compared to patients with solid tumors, patients with hematologic malignancies require more intensive medical care near the end of their lives [5-7]. They are less likely to receive hospice or inpatient palliative care services and have greater rates of emergency room (ER) visits, hospital admissions, intensive care unit admissions, extended hospital stays, blood and platelet transfusions, and in-hospital death when compared to patients with solid tumors. Additionally, compared to physicians who treat solid tumors, hematologic oncologists have been demonstrated to be more likely to continue systemic therapy in patients with poor performance status and limited life expectancy. Early palliative care, on the other hand, has been linked to fewer aggressive interventions, better symptom management, more conversations about goals of care, and improved patient and caregiver experiences [8]. Despite the recognized benefits of early palliative care, patients with advanced cancer frequently present to the emergency department (ED) because of acute symptoms, disease progression, or treatment-related complications [9]. 

Finding unmet palliative care needs and streamlining care pathways require an understanding of the causes of ED presentations and the results that follow [10]. Nevertheless, comparative data specifically assessing ED admissions among patients subsequently admitted to palliative care units is still scarce, despite established disparities in end-of-life care between hematologic and solid malignancies. Previous research has mostly looked at mixed oncology populations or focused on healthcare utilization without directly contrasting the emergency presentation traits of these two groups [11, 12]. 

Thus, the purpose of this study was to compare the short-term clinical outcomes and reasons for ED admission between patients admitted to an inpatient palliative care unit with solid tumors and those with hematologic malignancies. This study aims to provide evidence that could support the optimization of palliative care delivery for patients with advanced cancer by identifying disease-specific patterns and factors associated with adverse outcomes.

## Materials and methods

Study design and setting

This retrospective observational study was carried out at Princess Noorah Oncology Center, King Abdulaziz Medical City, a tertiary academic medical facility in Jeddah, Saudi Arabia, that has an inpatient palliative care unit and a specialized oncology ED. During the study period, the medical records of adult patients admitted straight from the ED to the inpatient palliative care unit were examined. The purpose of the study was to compare the short-term clinical outcomes and reasons for ED admission between patients with solid tumors and hematologic malignancies.

Study population

Adults (≥18 years) with a verified cancer diagnosis who were admitted straight from the ED to the inpatient palliative care unit were eligible to participate. Based on the type of cancer, patients were divided into two groups: solid tumors and hematologic malignancies, such as multiple myeloma, lymphoma, and leukemia. Patients who were admitted electively, had incomplete medical records, or were transferred from other hospital departments or outside healthcare facilities were not included.

Data collection

A standardized data collection form was used to extract data retrospectively from the hospital's electronic medical records. Age, sex, living status, and comorbidities were among the demographic factors. The type of cancer, the length of time since diagnosis, the disease's stage, and the metastatic status were all variables related to cancer. The main cause of ED presentation, the intensity of symptoms at presentation, and ED disposition were all considered ED variables. Dyspnea or respiratory symptoms, altered mental status or neurological symptoms, infection, fever, or sepsis, gastrointestinal symptoms or abdominal pain, bleeding, cardiovascular or fluid overload, urinary or renal symptoms, device- or procedure-related complications, and pain were all classified as reasons for ED presentation.

Outcome measures

The comparison of ED admission reasons for patients with solid tumors and those with hematologic malignancies who were admitted to the inpatient palliative care unit was the main objective. ED disposition, final hospital diagnosis, palliative care procedures carried out during admission, 30-day mortality, 30-day readmission, and the identification of factors independently associated with 30-day mortality were all considered secondary outcomes.

Ethical considerations

The study protocol was reviewed and approved by the Institutional Review Board of King Abdullah International Medical Research Center (KAIMRC) (Approval No. NRJ26/029/1). Written informed consent was obtained from all participants included in the study. All study procedures were conducted in accordance with the Declaration of Helsinki and applicable institutional guidelines.

Statistical analysis

Patients were categorized into two groups based on malignancy type. Continuous variables, including age and time to diagnosis, were tested for normality and summarized as mean ± SD or median (IQR) as appropriate. Comparisons between solid and hematologic malignancy groups were performed using the independent samples t-test for normally distributed variables and the Mann-Whitney U test for non-normally distributed variables. Categorical variables were compared between groups using the Chi-square test or Fisher’s exact test when expected cell counts were less than 5. Logistic regression analyses were performed to identify the predictors of 30-day mortality. Adjusted OR (aOR) with 95% CI was reported in the regression model. All statistical analyses were performed using the IBM SPSS Statistics version 26 software (IBM Corporation, Armonk, NY, USA), and a p-value less than 0.05 was considered statistically significant.

## Results

The baseline demographic and clinical characteristics of patients with solid and hematologic malignancies were summarised in Table 1. The mean age was significantly higher in the hematologic malignancy group compared to the solid tumor group (73.57 ± 15.19 vs 65.95 ± 14.92 years, p = 0.021), indicating that patients with hematologic malignancies were older at presentation. A significant difference was observed in cancer stage distribution (p < 0.001), with hematologic malignancy patients more frequently categorized as having recurrent/relapsed disease, while solid tumor patients were predominantly in the metastatic/advanced stage. Similarly, metastatic disease status differed significantly between groups (p < 0.001), reflecting a higher proportion of metastatic involvement among solid tumor patients. No significant differences were observed between groups in living situation, comorbidity profiles (cardiometabolic, cardiovascular, thromboembolic, neurologic/psychiatric, hepatic/infectious, renal/urologic), or readmission within 30 days (Table 1).

**Table 1:** Baseline demographic and clinical characteristics of the participants * Significant p value (<0.05); ¥: chi-square test; €: Fisher's exact test; ₶: t test; ₽: Mann-Whitney U test

Variable		Total (N=256) N (%)	Solid Tumor (n=233) n (%)	Hematologic Malignancy (n=23) n (%)	Test Statistics	p-value
Age, mean ± SD		66.64 ± 15.07	65.95 ± 14.92	73.57 ± 15.19	-2.331	0.021*^₶^
Time since diagnosis, median (IQR)		36.44 ± 95.11	22 (9–36)	29 (7–61)	-0.901	0.368^₽^
Living situation, n (%)						
Home		224 (87.5)	205 (88.0)	19 (82.6)	1.426	0.490^¥^
Assisted-living facility		28 (10.9)	24 (10.3)	4 (17.4)		
Nursing home		4 (1.6)	4 (1.7)	0 (0.0)		
Stage of cancer, n (%)						
Early stage		14 (5.5)	13 (5.6)	1 (4.3)	-	<0.001*^€^
Locally advanced		36 (14.1)	34 (14.6)	2 (8.7)		
Metastatic/advanced		190 (74.2)	183 (78.5)	7 (30.4)		
Recurrent/relapsed		16 (6.3)	3 (1.3)	13 (56.5)		
Metastatic disease, n (%)						
No		70 (27.3)	54 (23.2)	16 (69.6)	22.675	<0.001*^¥^
Yes		186 (72.7)	179 (76.8)	7 (30.4)		
Readmission within 30 days, n (%)						
No		103 (40.2)	93 (39.9)	10 (43.5)	0.111	0.739^¥^
Yes		153 (59.8)	140 (60.1)	13 (56.5)		
Comorbidities, n (%)						
Cardiometabolic disease						
No		92 (35.9)	85 (36.5)	7 (30.4)	0.332	0.564^¥^
Yes		164 (64.1)	148 (63.5)	16 (69.6)		
Cardiovascular disease						
No		192 (75.0)	178 (76.4)	14 (60.9)	-	0.101^¥^
Yes		64 (25.0)	55 (23.6)	9 (39.1)		
Thromboembolic disease						
No		217 (84.8)	197 (84.5)	20 (87.0)	-	1.000^€^
Yes		39 (15.2)	36 (15.5)	3 (13.0)		
Neurologic/Psychiatric disease						
No		239 (93.4)	219 (94.0)	20 (87.0)	-	0.187^€^
Yes		17 (6.6)	14 (6.0)	3 (13.0)		
Hepatic/Infectious disease						
No		227 (88.7)	206 (88.4)	21 (91.3)	-	1.000^€^
Yes		29 (11.3)	27 (11.6)	2 (8.7)		
Renal/Urological disease						
No		233 (91.0)	213 (91.4)	20 (87.0)	-	0.445^€^
Yes		23 (9.0)	20 (8.6)	3 (13.0)		
Other chronic conditions						
No		235 (91.8)	216 (92.7)	19 (82.6)	-	0.105^€^
Yes		21 (8.2)	17 (7.3)	4 (17.4)		

The most primary reasons for admission, including dyspnea, infection, gastrointestinal symptoms, cardiovascular causes, urinary causes, pain, bleeding, and device-related complications, did not differ significantly between hematologic and solid tumor patients. However, symptom severity at presentation (p = 0.001), with hematologic malignancy patients showing a higher proportion of mild presentations compared to solid tumor patients (Table 2).

**Table 2:** Comparison of primary reasons for ED admission and symptom burden between hematologic and solid tumor patients * Significant p value (<0.05); ¥: chi-square test; €: Fisher's exact test

Variable	Total (N=256) N (%)	Solid Tumor (n=233) n (%)	Hematologic Malignancy (n=23) n (%)	Test Statistics	p-value
Dyspnea/Respiratory symptoms, n (%)					
No	114 (44.5)	105 (45.1)	9 (39.1)	0.298	0.585^¥^
Yes	142 (55.5)	128 (54.9)	14 (60.9)		
Altered mental status/Neurological symptoms, n (%)					
No	199 (77.7)	180 (77.3)	19 (82.6)	0.347	0.556^¥^
Yes	57 (22.3)	53 (22.7)	4 (17.4)		
Infection/Fever/Sepsis, n (%)					
No	176 (68.8)	160 (68.7)	16 (69.6)	0.008	0.930^¥^
Yes	80 (31.3)	73 (31.3)	7 (30.4)		
Gastrointestinal symptoms/Abdominal pain, n (%)					
No	152 (59.4)	137 (58.8)	15 (65.2)	0.358	0.550^¥^
Yes	104 (40.6)	96 (41.2)	8 (34.8)		
Bleeding, n (%)					
No	230 (89.8)	212 (91.0)	18 (78.3)	-	0.068^€^
Yes	26 (10.2)	21 (9.0)	5 (21.7)		
Cardiovascular symptoms/Fluid overload, n (%)					
No	235 (91.8)	213 (91.4)	22 (95.7)	-	0.704^€^
Yes	21 (8.2)	20 (8.6)	1 (4.3)		
Urinary/Renal symptoms, n (%)					
No	218 (85.2)	198 (85.0)	20 (87.0)	-	1.000^€^
Yes	38 (14.8)	35 (15.0)	3 (13.0)		
Device-/Procedure-related symptoms, n (%)					
No	240 (93.8)	217 (93.1)	23 (100.0)	-	0.375^€^
Yes	16 (6.3)	16 (6.9)	0 (0.0)		
Pain, n (%)					
No	181 (70.7)	163 (70.0)	18 (78.3)	0.697	0.404^¥^
Yes	75 (29.3)	70 (30.0)	5 (21.7)		
Symptom severity at ED, n (%)					
Mild	3 (1.2)	1 (0.4)	2 (8.7)	-	0.001*^€^
Moderate	27 (10.5)	26 (11.2)	1 (4.3)		
Severe	226 (88.3)	206 (88.4)	20 (87.0)		
Palliative care procedures during stay					
No	176 (69)	158 (67.8)	18 (78.3)	1.010	0.315^¥^
Yes	79 (31)	74 (31.8)	5 (21.7)		

No statistically significant differences were observed between hematologic and solid tumor patients across final diagnosis categories, including respiratory infections, gastrointestinal/hepatobiliary infections, catheter-related issues, bleeding, and other causes (all p > 0.05). Similarly, ED disposition did not differ significantly between groups (p = 0.09), although nearly all patients in both groups were transitioned directly to palliative care units. Thirty-day survival outcomes were also comparable between groups, with no significant difference in mortality, survival, or unknown status (p = 0.573). Additionally, 30-day readmission rates were similar (p = 0.825) (Table 3).

**Table 3:** Comparison of hospital outcomes (ED disposition, mortality, readmission, palliative procedures).

Variable	Total (N=256) N (%)	Solid Tumor (n=233) n (%)	Hematologic Malignancy (n=23) n (%)	Test statistics	p-value
Final hospital diagnosis, n (%)					
Respiratory tract infection					
No	141 (55.1)	132 (56.7)	9 (39.1)	2.598	0.107¥
Yes	115 (44.9)	101 (43.3)	14 (60.9)		
Gastrointestinal/Hepatobiliary infection					
No	250 (97.7)	227 (97.4)	23 (100.0)	-	1.000€
Yes	6 (2.3)	6 (2.6)	0 (0.0)		
Catheter-related problems					
No	247 (96.5)	224 (96.1)	23 (100.0)	-	1.000€
Yes	9 (3.5)	9 (3.9)	0 (0.0)		
Bleeding					
No	220 (85.9)	202 (86.7)	18 (78.3)	-	0.340€
Yes	36 (14.1)	31 (13.3)	5 (21.7)		
Other causes					
No	121 (47.3)	110 (47.2)	11 (47.8)	0.003	0.955¥
Yes	135 (52.7)	123 (52.8)	12 (52.2)		
ED disposition, n (%)					
Direct palliative care	255 (99.6)	233 (100)	22 (95.7)	-	0.090€
Discharged	1 (0.4)	0	1 (4.3)		
30-day survival, n (%)					
Died	207 (80.9)	187 (80.3)	20 (87.0)	-	1.000€
Alive	40 (15.6)	37 (15.9)	3 (13.0)		
Unknown	9 (3.5)	9 (3.9)	0 (0.0)		
Re-admission within 30 days, n (%)					
No	103 (40.2)	93 (39.9)	10 (43.5)	0.111	0.825¥
Yes	153 (59.8)	140 (60.1)	13 (56.5)		

In multivariable logistic regression analysis, increasing age (aOR 1.037, 95% CI 1.008-1.066, p = 0.011) and severe symptom burden at ED presentation (aOR 8.165, 95% CI 3.086-21.605, p < 0.001) were independently associated with higher 30-day mortality. Living situation was also significantly associated with mortality (aOR 0.247, 95% CI 0.093-0.651, p = 0.005). No significant associations were observed for malignancy type, metastatic disease status, time since diagnosis, comorbidity burden, or individual presenting symptom categories. These findings suggest that patient age and symptom severity at presentation are the strongest determinants of short-term mortality among palliative care patients admitted through the emergency department (Table 4).

**Table 4:** Binary logistic regression analysis of factors associated with 30-day mortality. * Significant p-value

Variable	Adjusted OR	95% CI (Lower–Upper)	p-value
Male sex (vs Female)	0.539	0.233–1.245	0.148
Age (Per Year Increase)	1.037	1.008–1.066	0.011*
Hematologic malignancy (vs Solid tumor)	1.767	0.360–8.676	0.483
Time since diagnosis	1.000	0.995–1.006	0.891
Metastatic disease (Yes vs No)	1.149	0.450–2.935	0.772
Readmission within 30 days (Yes vs No)	1.996	0.925–4.306	0.078
Institutionalized living (vs Home)	0.247	0.093–0.651	0.005*
Palliative procedures during stay (Yes vs No)	0.503	0.225–1.122	0.093
Dyspnea/Respiratory presentation (Yes vs No)	0.413	0.147–1.159	0.093
Altered mental status/Neurological presentation (Yes vs No)	0.532	0.190–1.494	0.231
Infection/Fever/Sepsis presentation (Yes vs No)	1.414	0.591–3.381	0.436
Gastrointestinal/Abdominal pain presentation (Yes vs No)	1.012	0.391–2.624	0.98
Bleeding presentation (Yes vs No)	0.455	0.143–1.450	0.183
Cardiovascular/Fluid overload presentation (Yes vs No)	0.731	0.208–2.570	0.625
Urinary/Renal presentation (Yes vs No)	0.704	0.231–2.148	0.538
Device/Procedure-related presentation (Yes vs No)	0.522	0.116–2.342	0.396
Pain presentation (Yes vs No)	1.291	0.377–4.421	0.685
Number of comorbidities	0.844	0.682–1.046	0.121
Severe symptom burden (vs Mild/Moderate)	8.165	3.086–21.605	<0.001*

## Discussion

In this study, patients with solid tumors admitted to an inpatient palliative care unit were compared to those with hematologic malignancies in terms of the reasons for ED admission and short-term outcomes. The primary findings were that while patients with solid tumors were more likely to have metastatic disease, patients with hematologic malignancies were older and more likely to be admitted with recurrent or relapsed disease. On the other hand, the two groups' reasons for ED admission, hospital outcomes, 30-day mortality, and 30-day readmission were comparable. Furthermore, 30-day mortality was independently correlated with increasing age and severe symptom burden at ED presentation, but not with the type of cancer. Compared to patients with solid tumors, those with hematologic malignancies were noticeably older. The previous studies reported mixed findings; for instance, in a survivorship clinic cohort, patients with hematologic malignancies were younger than patients with solid malignancies: mean age 46.9 vs 56.8 years [13]. Several other studies show hematologic malignancies are common in older adults in absolute terms, but this does not imply older than solid tumors; about half to 60% of hematologic malignancy cases occur after age 65, often in the seventh and eighth decades [14-17]. Additionally, they were more likely to experience recurrent or relapsed disease, while patients with solid tumors were more likely to experience metastatic disease [18]. The primary reasons for ED admission did not differ significantly between the groups, according to the current study. Similar frequencies were observed for respiratory symptoms, infections, gastrointestinal symptoms, pain, neurological symptoms, urinary disorders, cardiovascular causes, and bleeding. A survey study found that the most frequent ED admissions were among patients with progressive cancer. The reasons for ED visits among cancer patients, such as pain, nausea and vomiting, and high fever, indicate that post-treatment follow-up and palliative care requirements are not adequately met [19]. Another cross-sectional study stated that the common types of cancer diagnosed at the ED were gastrointestinal and hematologic malignancies. Pain was the predominant complaint among oncology patients present in an ED [20]. This result contrasts with earlier research that found patients with hematologic malignancies used healthcare more frequently and received more aggressive end-of-life care, including higher rates of hospital admissions and ER visits. A retrospective cohort study found that in the last 30 days of life, hematologic patients were more likely to have ER visits, hospital admissions, >14 days of hospitalization, intensive care unit admissions, death, chemotherapy use, and targeted therapy use compared to patients with solid tumors [21]. The fact that the current study only included patients who had already been admitted to an inpatient palliative care unit may be one reason. The majority of patients in both groups had severe symptoms, even though the severity of symptoms varied considerably between groups, with a greater percentage of mild presentations among patients with hematologic malignancies. This finding should be interpreted with caution because the number of patients with hematologic malignancies was relatively small. A cross-sectional analysis study found that hematologic and solid tumor patients have significant symptom burden at the time of referral to palliative care services. Blood cancer patients may have unique concerns warranting targeted attention, including substantial drowsiness and tiredness. It has been stated in the study limitations that the sample was limited to cancer patients already referred to palliative care, making it likely that these patients are sicker than the cancer patient population at large, given what is known about the prevalence of late referrals to palliative and hospice care services [22].

The two groups' hospital outcomes were also similar; 30-day mortality, 30-day readmission, ED disposition, and final hospital diagnoses did not differ substantially. In contrast to the previous studies, which found that compared to patients with solid tumors, those with hematologic malignancies received more aggressive end-of-life care and were less likely to receive hospice or palliative care services [5, 10, 21, 23]. Since every patient in the current study had already been referred to specialized inpatient palliative care, the discrepancy might have been caused by the inclusion criteria. Higher 30-day mortality was found to be independently correlated with increasing age and severe symptom burden at ED presentation in the multivariable analysis. On the other hand, after adjustment, there was no correlation found between mortality and malignancy type, metastatic disease, time since diagnosis, comorbidity burden, or presenting symptoms. In an observational study, among ED patients with new-onset atrial fibrillation (AF), active cancer appears to be associated with 11-fold increased odds of 30-day mortality; new-onset AF may represent progressive organ dysfunction leading to an increased risk of short-term mortality in patients with cancer [24]. According to these results, clinical status at presentation has a stronger correlation with short-term mortality among patients admitted to a palliative care unit than cancer type.

The current research has several advantages. In particular, it compared solid and hematologic malignancies in patients who were admitted straight from the ER to an inpatient palliative care unit, a population that has received less attention. Additionally, there was less variation in data collection because all patients were treated in the same facility using a standardized method.

Limitations

There are a few limitations to be aware of. The results' generalizability is restricted by the retrospective single-center design. The ability to identify meaningful differences between groups may have been hampered by the comparatively small number of patients with hematologic malignancies. Additionally, factors like quality of life, performance status, the timing of palliative care referral, and ongoing anticancer treatment were unavailable and thus could not be assessed. To validate these results, more patients with hematologic malignancies must be included in future prospective multicenter studies. Research assessing quality-of-life outcomes, functional status, symptom assessment, and the timing of palliative care referral may enhance our knowledge of the variables affecting patients with advanced cancer who present to the ER and their outcomes.

## Conclusions

Patients with solid tumors and hematologic malignancies who were admitted from the ED to an inpatient palliative care unit had similar short-term outcomes and reasons for ED presentation despite differences in baseline disease characteristics. Patients with solid tumors were more likely to have metastatic disease, whereas patients with hematologic malignancies were older and more likely to have relapsed or recurrent disease. The strongest indicators of poor short-term outcomes were increasing age and severe symptom burden at ED presentation; malignancy type was not independently linked to 30-day mortality. These results imply that when it comes to predicting prognosis and making early decisions about palliative care, clinical status at presentation may be more significant than cancer type. Future multicenter prospective studies with larger cohorts of hematologic malignancies are required to confirm these results and improve palliative care pathways for patients with advanced cancer.
